# Effects of daily intake of cacao polyphenols, alone or with milk protein, on skeletal muscle mass in community-dwelling older adults: an exploratory 16-week randomized, open-label, parallel-group trial

**DOI:** 10.1186/s40795-026-01445-7

**Published:** 2026-08-03

**Authors:** Tetsuzen Rin, Midori Natsume, Kaori Iwasawa, Chiharu Iwasaka, Akane Haseda, Hiroyo Kagami-Katsuyama, Naoyuki Honma, Jun Nishihira

**Affiliations:** 1 Meiji Holdings Co., Ltd, 2-4-16 Kyobashi, Chuo-ku, Tokyo, 104-0031 Japan; 2 Meiji Co., Ltd, 2-2-1 Kyobashi, Chuo-ku, Tokyo, 104-8306 Japan; 3 Hokkaido Information University, 59-2 Nishi-Nopporo, Ebetsu, Hokkaido 069-8585 Japan

**Keywords:** Cacao flavanols, Milk protein, Older adults, Skeletal muscle mass, Body composition

## Abstract

**Background:**

Evidence on food-based strategies to maintain muscle-related body composition in older adults under habitual lifestyle conditions is limited. Cacao flavanols may influence vascular and redox pathways, whereas milk protein provides amino acids relevant to muscle protein synthesis. We examined whether daily high-cacao chocolate, alone or with a protein-enriched milk beverage, affected body composition in community-dwelling older adults.

**Methods:**

This exploratory 16-week randomized, open-label, parallel-group trial included adults aged 60–79 years. Participants were allocated 1:1:1 to no intake (NI), high-cacao chocolate (HC; 15 g/day), or HC plus a protein-enriched milk beverage (HC+MILK; 200 mL/day). Body composition was assessed by multifrequency bioelectrical impedance analysis (BIA) at baseline, week 8, and week 16. Longitudinal models evaluated observed values and changes from baseline, and pairwise comparisons were adjusted using Tukey’s method. The per-protocol set was the primary efficacy population.

**Results:**

Of 240 randomized participants, 219 were included in the per-protocol set (NI, 76; HC, 72; HC+MILK, 71). At week 16, soft lean mass changed by − 0.24, + 0.21, and + 0.25 kg in NI, HC, and HC+MILK, respectively. Compared with NI, the change was + 0.45 kg with HC (95% CI 0.06 to 0.83; *P* = 0.015) and + 0.49 kg with HC+MILK (0.10 to 0.88; *P* = 0.005). Fat-free mass, skeletal muscle mass, and skeletal muscle index showed consistent significant differences versus NI, whereas HC+MILK did not differ from HC. No intervention-specific effect was observed for physical-function outcomes, and no product-related adverse events occurred.

**Conclusions:**

Over 16 weeks, HC and HC+MILK showed statistically significant favorable changes in BIA-derived muscle-related indices compared with NI, without a clear incremental benefit of adding MILK. These findings suggest a potential benefit for maintaining BIA-estimated muscle mass under habitual activity conditions, although the clinical relevance remains uncertain because no intervention-specific functional benefit was detected and changes in exercise type and intensity were not assessed comprehensively.

**Trial registration:**

UMIN000054576 (June 6, 2024).

**Supplementary Information:**

The online version contains supplementary material available at https://doi.org/10.1186/s40795-026-01445-7.

## Background

In Japan, extending healthy life expectancy has become a national priority [1]. Internationally, the World Health Organization’s Integrated Care for Older People framework promotes person-centred assessment and care pathways to identify and manage declines in intrinsic capacity among older people [2]. Sarcopenia is characterized by a reduction in muscle mass and function and is associated with falls, disability, and mortality [3]. In 2019, the Asian Working Group for Sarcopenia (AWGS) recommended a multidomain assessment of muscle mass, strength, and physical performance [4]. Longitudinal data revealed that muscle mass declines at around 60 years of age and decreases by approximately 0.6–1.0% annually after 70 years, based on dual-energy X-ray absorptiometry (DXA) and other related modalities [5, 6]. Globally, healthy life expectancy has been adopted as a key monitoring indicator, and the World Health Organization’s Decade of Healthy Ageing 2021–2030 has focused on ensuring longer and healthier lives for everyone [7].

A combination of resistance exercise and adequate protein intake is recommended to preserve or increase muscle mass in older adults [8]. However, in real-world settings, including among community-dwelling older adults and those receiving outpatient care, comorbidities, pain, fear of falling, logistical burdens, appetite loss, and chewing or swallowing difficulties can impede sustained exercise and adequate protein intake. Nutritional strategies that can be implemented without a prescribed exercise program therefore merit further investigation.

Age-related anabolic resistance emphasizes the importance of protein quantity, quality (including essential amino acids and leucine), and timing or distribution [9, 10]. Older adults may therefore require adequate protein at each meal; however, appetite decline, impaired mastication or swallowing, and limited intake capacity can make this difficult. In older Japanese women, higher protein intake combined with higher dietary total antioxidant capacity was associated with a lower prevalence of frailty [11]. Milk protein was selected as the supplemental protein source because a randomized placebo-controlled trial in healthy older adults reported that low-dose milk-protein supplementation combined with low-to-moderate-intensity exercise increased muscle mass [12]. The ready-to-drink formulation used in the present trial also allowed a standardized dose of 14.8 g of protein to be provided in a practical 200-mL serving. This selection was not intended to imply superiority over other protein sources, which were not compared in this trial.

Cacao is a well-recognized source of flavanols, and high-cocoa products provide antioxidant compounds [13]. Cacao flavanols help maintain endothelium-dependent vasodilation [14], and randomized trials have reported improvements in endothelial function and cardiovascular risk markers [15]. In older adults, cacao flavanols administered with an oral nutritional supplement acutely increased muscle microvascular blood volume [16]. Human skeletal muscle studies have also reported activation of nuclear factor erythroid 2-related factor 2 pathways [17], and a high-flavanol cacao supplement improved mobility and quality of life in healthy older adults [18].

Nevertheless, randomized trials investigating the regular intake of antioxidant-rich cacao products, alone or combined with protein supplementation, without a mandatory exercise program remain scarce. We therefore conducted an exploratory randomized trial to examine whether 16 weeks of daily high-cacao chocolate, alone or combined with a milk-protein beverage, affected body-composition indices in community-dwelling older adults.

## Materials and methods

### Trial design

Given the limited evidence, we conducted a 16-week exploratory, randomized, open-label, parallel-group trial (three arms; 1:1:1) without a prescribed structured exercise program in community-dwelling older adults aged 60–79 years. The assessment schedule was prespecified during protocol development. Week 8 was selected as an intermediate assessment with reference to the cumulative cacao-polyphenol exposure in a previous 4-week intervention using 25 g/day of high-cacao chocolate [19], whereas week 16 was selected as the primary assessment, informed in part by the cumulative milk-protein exposure in a previous 6-month trial using 10 g/day of milk protein in older adults [12]. These comparisons were used as pragmatic design references and did not imply biological equivalence across studies. Feasibility and participant burden were also considered. Participants were assigned to NI, HC, or HC+MILK. No changes were made to the methods after trial commencement, including the eligibility criteria.

### Setting and timeline

Clinical visits were conducted at baseline (July–August 2024), week 8 (September–October), and week 16 (November–December). Recruitment was publicized from June 6 to 10, 2024, via community posts and web notices, and enrolment was conducted via fax. Outcome assessments were scheduled for days 56 and 112 after initiating intake (± 14 days).

### Visit schedule and participant instructions

Outcomes were assessed at each visit. Participants were instructed to refrain from consuming the study foods on visit days, avoid major lifestyle changes and initiation of new medications, maintain their habitual physical activity, and not initiate a new structured exercise program during the study. During the study, participants were also instructed not to consume additional chocolate containing ≥ 70% cacao or products labeled as high protein that provided ≥ 10 g protein per package. They were also asked to complete daily study-product adherence diaries documenting study-product intake, prohibited-food intake, health changes, non-routine medication use, medical visits, and step counts.

### Participants

The inclusion criteria were age 60–79 years as of April 1, 2024; independent living; provision of written informed consent; ability to attend scheduled visits and complete questionnaires and study-product adherence diaries; and agreement to comply with the study’s dietary restrictions, including avoidance of additional chocolate containing ≥ 70% cacao and products labeled as high protein that provided ≥ 10 g protein per package.

The exclusion criteria included non-independent ambulation or dependency in basic activities of daily living; active severe major-organ disease or an early postoperative period after major surgery; treatment for neurodegenerative or neuromuscular disorders; implanted medical devices that could interfere with safety or measurements; allergy to milk, soy, or cacao; recent blood donation or participation in another clinical trial within predefined windows; habitual intake of ≥ 40 g/day of dark or bitter chocolate or ≥ 100 g/day of milk chocolate on approximately 3 or more days per week; extreme alcohol or tobacco use; markedly unstable lifestyle; or other conditions judged unsuitable by the investigator. Recent weight loss, malnutrition, frailty, low muscle mass, and sarcopenia were not diagnosis-based exclusion criteria.

### Interventions

The NI group did not receive study food. The HC group received 15 g/day of HC (total product weight; three 5-g pieces), providing 6.7 g/day of carbohydrate. The HC+MILK group received the same 15-g/day serving of HC plus 200 mL/day of a commercially available protein-enriched, low-fat milk beverage (MILK; 14.8 g protein per serving). The intervention lasted 16 consecutive weeks. Both study foods were manufactured by Meiji Co., Ltd. and supplied by Meiji Holdings Co., Ltd. to Hokkaido Information University, which distributed the assigned products to participants.

The daily energy, macronutrient, sodium chloride equivalent, and cacao-derived constituent contents of the study foods are summarized in Table 1. Total cacao polyphenols were quantified using the Folin–Ciocalteu method and expressed as (−)-epicatechin equivalents using (−)-epicatechin (Cat. No. E1753-1G; Sigma-Aldrich, Merck KGaA, Darmstadt, Germany) as the reference standard [20, 21]. Cacao flavanols (degree of polymerization 1–7) were quantified according to AOAC Official Method 2020.05 using high-performance liquid chromatography with fluorescence detection [22]. Major procyanidins, including (−)-epicatechin, (+)-catechin, procyanidin B2, procyanidin B5, procyanidin C1, and cinnamtannin A2, were quantified as (−)-epicatechin equivalents by internal reverse-phase high-performance liquid chromatography with ultraviolet detection at 280 nm, based on the method of Natsume et al. with minor modifications [23]. Cacao-derived constituents in MILK were not determined.

**Table 1:** Composition of the study foods

Component	Unit	HC (15 g of chocolate/day)	MILK (200 mL/day)
Energy	kcal	85	104
Protein	g	1.6	14.8
Fat	g	6.1	0.4
Carbohydrate	g	6.7	10.4
Salt (sodium chloride) equivalent	g	0.0	0.3
Total cacao polyphenols	mg	381	Not determined
Cacao flavanols	mg	105	Not determined
Procyanidins	mg	62	Not determined

*HC* high-cacao chocolate. Values represent the daily nutrient and cacao-constituent amounts provided by the study foods, not total dietary intake

### Outcomes

Within the exploratory framework, body composition was prespecified as the primary outcome domain and comprised eight indices: body weight, body fat percentage, body mass index (BMI), soft lean mass (SLM), fat-free mass (FFM), skeletal muscle mass (SMM), skeletal muscle index (SMI), and extracellular water/total body water (ECW/TBW). All eight indices are reported transparently, while the narrative interpretation focuses on representative muscle-related outcomes, particularly SLM and SMI. Observed values and changes from baseline were analyzed at weeks 8 and 16. Week 8 was the prespecified intermediate assessment, and week 16 was the prespecified primary assessment. Secondary outcomes included physical performance (4-m walking time, five-times sit-to-stand time, ZaRits power, speed, and balance, and handgrip strength); step counts; vital signs (systolic and diastolic blood pressure); blood biomarkers of inflammation (high-sensitivity C-reactive protein [hs-CRP]), lipid profile (total cholesterol [TC], low-density lipoprotein cholesterol [LDL-C], high-density lipoprotein cholesterol [HDL-C], triglycerides [TG], and the LDL/HDL ratio), glucose levels (fasting plasma glucose and hemoglobin A1c [HbA1c]), oxidative stress (serum 8-hydroxy-2′-deoxyguanosine [8-OHdG]), and routine safety (hematology and liver- and kidney-function variables); and frailty-related questionnaire measures. Dietary intake was assessed at baseline and week 16 as a potential dietary confounder, as described in Sect. 2.8. Physical-function outcomes were prespecified to explore whether changes in muscle-related body composition were accompanied by changes in function relevant to older adults. No changes to the prespecified outcomes were made after trial commencement.

### Measurements

#### Body composition

Body composition was assessed between 09:00 and 13:30 using multifrequency bioelectrical impedance analysis (BIA) with an InBody 770 analyzer (InBody Co., Ltd.). BIA was selected as a feasible method for repeated assessments at three timepoints in this relatively large, community-based exploratory trial. DXA was not included in the prespecified protocol, and bone mineral content was therefore not assessed. Follow-up assessments for each participant were generally scheduled at approximately the same time of day as the baseline assessment. Participants were instructed to avoid vigorous exercise causing sweating and alcohol from the day before testing, to refrain from breakfast on the test day, and to wear light clothing. Water intake was unrestricted before arrival but was prohibited from registration until completion of testing. Participants were instructed to void approximately 30 min before the body-composition assessment. The assessment was conducted after all other examinations, without a fixed rest period, and participants refrained from consuming the study foods until completion of testing. Recorded parameters included body weight, body fat percentage, BMI, SLM, FFM, SMM, SMI, and ECW/TBW.

#### Physical function

Physical-function outcomes were measured using standardized protocols. Gait speed was assessed over a 4-m straight path without a run-up, based on a single trial, using a Witty timing system (Microgate S.r.l., Bolzano, Italy). The five-times sit-to-stand test was conducted using a chair 40–43 cm high, and the time required to complete five rises was measured. Power, speed, and balance were assessed using a ZaRits BM-220 device (Tanita Corporation, Tokyo, Japan) according to the manufacturer’s specifications, and device calibration was verified. Handgrip strength was measured with a digital Smedley dynamometer (TKK 5401; Takei Scientific Instruments Co., Ltd., Niigata, Japan); participants performed two attempts per side, and the maximum value was recorded. General exercise habits and recent physical activity were assessed at baseline. In addition, step counts were assessed at baseline, week 8, and week 16 using an activity monitor (UW-201NFC; A&D Company, Limited, Tokyo, Japan). Participants recorded daily steps from waking to bedtime, excluding periods such as bathing or swimming, for three consecutive days specified for each visit, and the mean of the available measurement days was used for each timepoint.

#### Blood analyses

Blood samples were collected from participants, and serum, plasma, or whole-blood specimens were used as appropriate for each assay. The measured or derived variables included white blood cell count, red blood cell count, hemoglobin, hematocrit, platelet count, aspartate aminotransferase, alanine aminotransferase, γ-glutamyl transpeptidase, alkaline phosphatase, lactate dehydrogenase, Fibrosis-4 index, blood urea nitrogen, creatinine, uric acid, high-sensitivity C-reactive protein (hs-CRP), total cholesterol (TC), low-density lipoprotein cholesterol (LDL-C), high-density lipoprotein cholesterol (HDL-C), triglycerides (TG), LDL/HDL ratio, fasting plasma glucose, hemoglobin A1c (HbA1c), and serum 8-hydroxy-2′-deoxyguanosine (8-OHdG). Plasma was used for fasting glucose, whole blood for HbA1c and hematology, and serum for the relevant chemistry and 8-OHdG assays; the LDL/HDL ratio and Fibrosis-4 index were calculated. Routine analyses other than 8-OHdG were performed by Sapporo Clinical Laboratory, Inc., using its standard same-day processing and transport procedures. Serum samples for 8-OHdG were prepared at the Health Information Science Research Center, Hokkaido Information University, stored at − 80 °C until analysis, and measured at Meiji Holdings Co., Ltd. using a high-sensitivity 8-OHdG ELISA kit (Japan Institute for the Control of Aging, Shizuoka, Japan).

### Dietary assessment

Dietary intake was assessed at baseline and week 16 using the Brief-type self-administered Diet History Questionnaire (BDHQ), a fixed-portion self-administered diet-history questionnaire that estimates habitual intake during the preceding month from reported frequencies of food and beverage consumption. Daily energy and nutrient intakes were estimated using validated algorithms developed for Japanese adults [24]. Validation against 16-day semi-weighed dietary records supports the ability of the BDHQ to rank individuals for many nutrients, although absolute energy and protein estimates remain subject to self-report and estimation error. Comprehensive food records and 24-h dietary recalls were not collected.

### Sample size

Given the exploratory nature of the trial and the limited prior data available for a formal efficacy calculation based on a clinically meaningful change, we targeted 80 participants per group (total *n* = 240) based on feasibility. No interim analyses were performed.

### Randomization: sequence, concealment, and implementation

Randomization was implemented at the Media Education Center, Kyushu Institute of Information Sciences (Ebetsu, Hokkaido, Japan) by an allocation manager and independent allocation staff. Allocation was performed using permuted blocks (1:1:1) stratified by sex (male/female) and age (< 65, < 70, <75, and < 80 years). The randomized sequence was computer-generated. Allocation was concealed using an access-controlled sequence list, and the block size remained undisclosed to preserve allocation independence. The staff responsible for registration and allocation had no role in outcome measurement. After site registration, central randomization was used to assign the participants to groups.

### Blinding

This trial was open-label. The outcome assessors were blinded to the group allocation. Statistical analyses were performed on masked datasets with group codes revealed after completion of the analysis. A double-blind placebo-controlled design was not implemented because producing a sensory-matched placebo for HC and an isocaloric control while maintaining a comparable protein framework across study arms was not feasible considering study constraints.

### Data handling and missing data

Missingness was handled under the missing-at-random assumption within the linear mixed model framework. No additional imputation was applied, except for censoring at the limits of detection where relevant.

A portion of the collected serum was stored at Meiji Holdings Co., Ltd. The storage duration was set at 5 years after study completion or 3 years after manuscript publication, whichever occurred later, after which the samples were to be appropriately destroyed. Paper-based records would be digitized after 5 years, and all materials would be handled and discarded to prevent individual identification. No personally identifiable information (e.g., name, address, or contact details) was provided to any testing facility.

### Adherence

Participants recorded daily whether they consumed the full assigned amount of each study product as “consumed all” or “did not consume.” The diaries also captured prohibited-food intake, health changes, non-routine medication use, medical visits, and step counts; they were used to monitor adherence and were not comprehensive dietary records. Adherence was calculated as the number of days recorded as “consumed all” divided by the 112 planned intervention days, multiplied by 100. Major protocol deviations were defined as violations of the eligibility criteria, adherence < 80%, serious violations of concomitant-medication restrictions, or discontinuation because of safety concerns.

### Statistical analysis

The per-protocol set (PPS) was used for the primary efficacy analyses, and the full analysis set (FAS) was used for sensitivity analyses. Longitudinal linear mixed models included a participant-specific random intercept. For observed follow-up values, fixed effects were the corresponding baseline value, time, group, and the time-by-group interaction. Models of changes from baseline included time, group, and their interaction. Week 16 was the prespecified primary assessment, and week 8 was the prespecified intermediate assessment. Estimated marginal means and pairwise contrasts were adjusted using Tukey’s method; for the change analyses, simultaneous confidence intervals and adjusted P values reflected comparisons among the six time-by-group estimated changes. Baseline age, height, body weight, percent body fat, and BMI were compared using one-way analysis of variance, and sex was compared using the chi-square test. Two-sided P values < 0.05 were considered statistically significant, including Tukey-adjusted P values for post-hoc comparisons. Baseline-adjusted standardized mean differences were estimated separately at weeks 8 and 16 using analysis-of-covariance models containing group and the corresponding baseline value; contrasts were standardized using the residual standard deviation of each model. Analyses were conducted using SPSS version 25 (IBM Corp., Armonk, NY, USA) and R version 4.4.2 (R Foundation for Statistical Computing, Vienna, Austria).

### Ethics

The study complied with the Declaration of Helsinki and was approved by the Research Ethics Committee of Hokkaido Information University (approval number 2024-03; May 23, 2024). Adverse events (AEs) and serious adverse events (SAEs) were defined according to International Council for Harmonisation Good Clinical Practice guidelines. Events were collected during study visits or from spontaneous participant reports and were assessed for severity and relationship to the study products. The trial was registered in UMIN-CTR (UMIN000054576). Reporting followed the CONSORT 2025 statement [25]. Written informed consent was obtained from all participants. Data were anonymized and analyzed using de-identified datasets.

## Results

### Participant flow

**Fig. 1:**
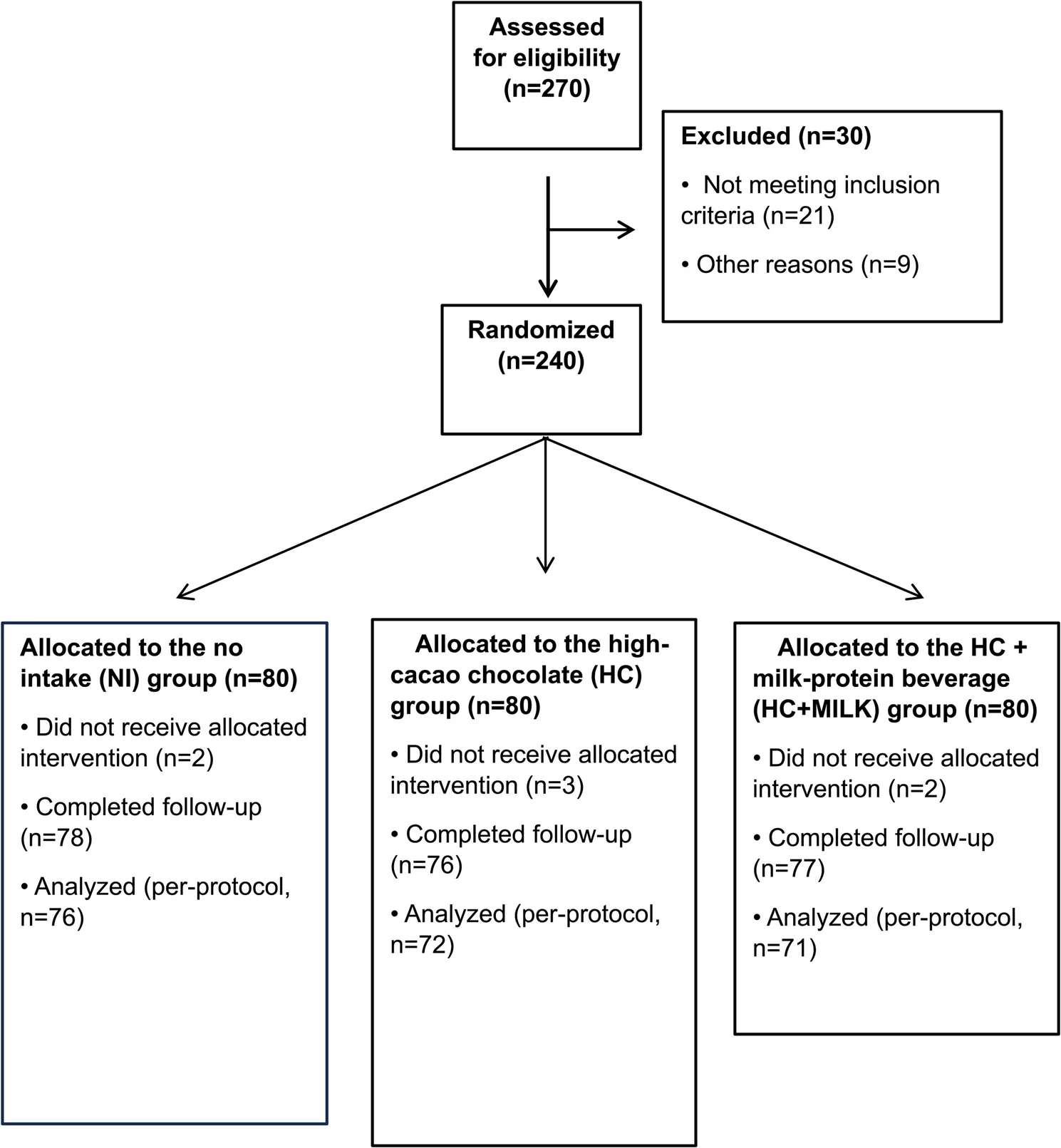
CONSORT flow diagram. Screening (*n* = 270), randomization (*n* = 240), intervention start (*n* = 236), withdrawal (*n* = 5), study completion (*n* = 231), and analysis. The full analysis set (FAS, *n* = 233) included all randomized participants with at least one post-baseline assessment; the per-protocol set (PPS, *n* = 219) excluded those with major protocol deviations. Group sizes at each stage are indicated for no intake (NI), high-cacao chocolate (HC), and high-cacao chocolate plus milk-protein beverage (HC+MILK). CONSORT, Consolidated Standards of Reporting Trials; FAS, full analysis set; PPS, per-protocol set

We randomized 240 participants, of whom 236 commenced the intervention (four missed the pre-intake visit); however, three participants discontinued the intervention before observation (HC, 2; HC+MILK, 1) and two discontinued after allocation (HC, 1; HC+MILK, 1). A total of 231 participants completed the study (NI, 78; HC, 76; HC+MILK, 77). The FAS included 233 participants, and the PPS included 219 participants (NI, 76; HC, 72; HC+MILK, 71). Figure 1 shows the CONSORT flow diagram. The baseline characteristics of the participants are summarized in Table 2. The trial was completed as planned after the prespecified 16-week follow-up and was not stopped early.

**Table 2:** Baseline characteristics by group

Variable	NI (*n* = 76)	HC (*n* = 72)	HC+MILK (*n* = 71)	*P* value
Age (y)	66.1 ± 5.0	65.9 ± 4.7	65.9 ± 4.9	0.952
Women, n (%)	57 (75.00)	53 (73.61)	49 (69.01)	0.699
Men, n (%)	19 (25.00)	19 (26.39)	22 (30.99)	—
Height (cm)	157.9 ± 7.5	160.0 ± 8.6	159.1 ± 8.0	0.258
Weight (kg)	55.0 ± 11.8	54.3 ± 10.1	55.2 ± 10.0	0.854
BMI (kg/m²)	22.0 ± 3.8	21.1 ± 2.9	21.7 ± 3.0	0.251
Percent body fat (%)	27.4 ± 7.6	25.8 ± 7.5	26.5 ± 8.2	0.450
SLM (kg)	37.3 ± 7.4	37.6 ± 7.2	38.0 ± 7.5	—
FFM (kg)	39.5 ± 7.8	39.9 ± 7.6	40.3 ± 7.8	—
SMM (kg)	21.1 ± 4.7	21.4 ± 4.6	21.7 ± 4.7	—
SMI (kg/m²)	6.3 ± 1.0	6.2 ± 0.9	6.3 ± 1.0	—
ECW/TBW (ratio)	0.40 ± 0.01	0.40 ± 0.01	0.40 ± 0.01	—
Energy intake (kcal/day)	1578.18 ± 406.48	1654.87 ± 462.15	1613.52 ± 552.88	—
Energy intake (kcal/kg/day)	29.8 ± 9.5	31.3 ± 9.9	30.2 ± 12.8	—
Protein intake (g/day)	65.47 ± 18.65	70.44 ± 21.82	64.92 ± 22.36	—
Protein intake (g/kg/day)	1.25 ± 0.44	1.34 ± 0.50	1.23 ± 0.54	—
Fat (g/day)	51.62 ± 13.90	53.42 ± 15.19	52.04 ± 19.36	—
Carbohydrate (g/day)	202.36 ± 61.43	213.04 ± 72.66	208.73 ± 81.40	—
Frailty category based on the 25-item frailty checklist, n (%)				
Robust	34 (44.74)	37 (51.39)	34 (47.89)	—
Prefrail	27 (35.53)	29 (40.28)	27 (38.03)	—
Frail	15 (19.74)	6 (8.33)	10 (14.08)	—
Low SMI, n/N (%)	27/76 (35.53)	28/71 (39.44)	23/71 (32.39)	—
Gait speed < 1.0 m/s, n (%)	3 (3.95)	3 (4.17)	2 (2.82)	—
Low handgrip strength, n (%)	16 (21.05)	16 (22.22)	19 (26.76)	—

*Values are presented as means ± SDs or numbers (%). P values are shown for the baseline comparisons performed for age, sex, height, body weight, BMI, and percent body fat; other variables are presented descriptively. Low SMI was defined using the AWGS 2019 criterion, and gait speed < 1.0 m/s is shown descriptively. One participant lacked a baseline SMI measurement

*BMI* body mass index, *SLM* soft lean mass, *FFM* fat-free mass, *SMM* skeletal muscle mass, *SMI* skeletal muscle index, *ECW/TBW* extracellular water/total body water, *NI* no intake, *HC* high cacao, *HC+MILK* high cacao plus milk-protein beverage

### Baseline data

Baseline age, height, body weight, percent body fat, BMI, and sex did not differ significantly among groups (P = 0.952, 0.258, 0.854, 0.450, 0.251, and 0.699, respectively; Table 2). Mean estimated protein intake was 1.25 ± 0.44, 1.34 ± 0.50, and 1.23 ± 0.54 g/kg/day in NI, HC, and HC+MILK, respectively (Table 2). According to the baseline 25-item frailty checklist, 105 of 219 participants were classified as robust, 83 as prefrail, and 31 as frail. Descriptively, 78 of 218 participants met the AWGS 2019 low-SMI criterion, 8 of 219 had gait speed < 1.0 m/s, and 51 of 219 met the applied low-handgrip-strength criterion (Table 2).

### Outcomes and estimation

Primary analyses were performed in the PPS (n = 219), with FAS sensitivity analyses yielding a similar overall pattern. Mean adherence was 99.18% for HC in the HC group and 98.89% for HC and 99.32% for MILK in the HC+MILK group. The assigned study products provided 85 kcal/day in HC and 189 kcal/day in HC+MILK; thus, HC+MILK provided 104 kcal/day more than HC. In observed-value models, significant time-by-group interactions were identified for body weight (P = 0.004), BMI (P = 0.006), SLM (P = 0.001), FFM (P = 0.001), SMM (P = 0.001), and SMI (P = 0.003), but not for percent body fat or ECW/TBW.

At week 16, the representative outcomes SLM and SMI changed by − 0.24, + 0.21, and + 0.25 kg for SLM and by − 0.09, − 0.01, and − 0.01 kg/m² for SMI in NI, HC, and HC+MILK, respectively (Table 3). The HC − NI and HC+MILK − NI differences were + 0.45 kg (95% CI 0.06 to 0.83; P = 0.015) and + 0.49 kg (0.10 to 0.88; P = 0.005) for SLM, and + 0.08 kg/m² (0.01 to 0.15; P = 0.024) and + 0.08 kg/m² (0.01 to 0.16; P = 0.016) for SMI. FFM and SMM showed directionally consistent findings: changes were − 0.24, + 0.21, and + 0.28 kg for FFM and − 0.08, + 0.16, and + 0.21 kg for SMM (Table 3), with HC − NI and HC+MILK − NI differences of + 0.45 kg (0.04 to 0.86; P = 0.021) and + 0.52 kg (0.11 to 0.93; P = 0.004) for FFM, and + 0.24 kg (0.02 to 0.46; P = 0.026) and + 0.30 kg (0.07 to 0.52; P = 0.002) for SMM. The HC+MILK − HC differences were small and nonsignificant: +0.05 kg for SLM (− 0.35 to 0.44; P = 0.999), + 0.07 kg for FFM (− 0.35 to 0.49; P = 0.997), + 0.06 kg for SMM (− 0.17 to 0.29; P = 0.979), and + 0.00 kg/m² for SMI (− 0.07 to 0.08; P = 1.000). Baseline-adjusted standardized mean differences for HC − NI and HC+MILK − NI ranged from 0.50 to 0.56 and from 0.54 to 0.64, respectively, whereas those for HC+MILK − HC ranged from 0.05 to 0.12 (Additional file 6).

**Table 3:** Body composition outcomes at baseline, week 8, and week 16

Time	NI (*n* = 76)	HC (*n* = 72)	HC+MILK (*n* = 71)	NI vs. HC (*P*-value)	NI vs. HC+MILK (*P*-value)	HC vs. HC+MILK (*P*-value)
(a) Weight (kg)						
Baseline (Week 0)	54.78 ± 0.10 [54.58, 54.98]	54.78 ± 0.10 [54.57, 54.98]	54.78 ± 0.10 [54.58, 54.99]	0.999	0.999	0.999
Week 8	54.82 ± 0.10 [54.62, 55.01]	55.02 ± 0.10 [54.82, 55.22]	55.27 ± 0.10 [55.07, 55.48]	0.895	**0.043**	0.720
Week 16	54.95 ± 0.10 [54.75, 55.14]	55.32 ± 0.10 [55.11, 55.52]	55.56 ± 0.11 [55.35, 55.76]	0.200	**0.001**	0.793
Δ Week 8 (change from baseline)	0.04 ± 0.12 [-0.21, 0.28]	0.24 ± 0.13 [-0.01, 0.49]	0.50 ± 0.13 [0.25, 0.75]	0.849	0.096	0.708
Δ Week 16 (change from baseline)	0.17 ± 0.12 [-0.08, 0.41]	0.54 ± 0.13 [0.29, 0.79]	0.78 ± 0.13 [0.52, 1.03]	0.277	**0.009**	0.784
(b) BMI (kg/m²)						
Baseline (Week 0)	21.58 ± 0.04 [21.51, 21.66]	21.58 ± 0.04 [21.50, 21.66]	21.58 ± 0.04 [21.50, 21.66]	0.999	0.999	0.999
Week 8	21.61 ± 0.04 [21.54, 21.69]	21.67 ± 0.04 [21.59, 21.75]	21.77 ± 0.04 [21.69, 21.85]	0.976	0.103	0.720
Week 16	21.66 ± 0.04 [21.59, 21.74]	21.79 ± 0.04 [21.71, 21.86]	21.90 ± 0.04 [21.82, 21.98]	0.418	**0.001**	0.603
Δ Week 8 (change from baseline)	0.03 ± 0.05 [-0.06, 0.12]	0.09 ± 0.05 [0.00, 0.19]	0.19 ± 0.05 [0.10, 0.29]	0.936	0.164	0.706
Δ Week 16 (change from baseline)	0.08 ± 0.05 [-0.01, 0.18]	0.21 ± 0.05 [0.11, 0.30]	0.31 ± 0.05 [0.22, 0.41]	0.444	**0.011**	0.645
(c) Percent body fat (%)						
Baseline (Week 0)	26.60 ± 0.13 [26.34, 26.86]	26.58 ± 0.14 [26.31, 26.85]	26.59 ± 0.14 [26.32, 26.85]	0.999	0.999	0.999
Week 8	27.35 ± 0.13 [27.09, 27.60]	27.00 ± 0.14 [26.73, 27.27]	27.19 ± 0.14 [26.92, 27.46]	0.662	0.996	0.987
Week 16	27.24 ± 0.13 [26.98, 27.50]	26.94 ± 0.14 [26.67, 27.21]	27.09 ± 0.14 [26.82, 27.36]	0.816	0.997	0.998
Δ Week 8 (change from baseline)	0.75 ± 0.16 [0.43, 1.06]	0.42 ± 0.17 [0.09, 0.75]	0.60 ± 0.17 [0.27, 0.93]	0.727	0.989	0.976
Δ Week 16 (change from baseline)	0.64 ± 0.16 [0.32, 0.96]	0.36 ± 0.17 [0.03, 0.69]	0.50 ± 0.17 [0.17, 0.83]	0.839	0.991	0.993
(d) SLM (kg)						
Baseline (Week 0)	37.61 ± 0.08 [37.47, 37.76]	37.62 ± 0.08 [37.47, 37.77]	37.63 ± 0.08 [37.47, 37.78]	0.999	0.999	0.999
Week 8	37.25 ± 0.08 [37.10, 37.40]	37.57 ± 0.08 [37.42, 37.73]	37.62 ± 0.08 [37.46, 37.78]	0.080	**0.024**	0.999
Week 16	37.38 ± 0.08 [37.23, 37.53]	37.83 ± 0.08 [37.67, 37.98]	37.88 ± 0.08 [37.72, 38.03]	**0.001**	< 0.001	0.999
Δ Week 8 (change from baseline)	-0.36 ± 0.09 [-0.55, -0.18]	-0.05 ± 0.10 [-0.24, 0.15]	0.00 ± 0.10 [-0.19, 0.20]	0.184	0.080	0.999
Δ Week 16 (change from baseline)	-0.24 ± 0.09 [-0.42, -0.05]	0.21 ± 0.10 [0.01, 0.40]	0.25 ± 0.10 [0.06, 0.45]	**0.015**	**0.005**	0.999
(e) FFM (kg)						
Baseline (Week 0)	39.89 ± 0.08 [39.73, 40.05]	39.90 ± 0.08 [39.73, 40.06]	39.90 ± 0.08 [39.74, 40.06]	0.999	0.999	0.999
Week 8	39.52 ± 0.08 [39.36, 39.68]	39.85 ± 0.08 [39.69, 40.02]	39.91 ± 0.08 [39.74, 40.07]	0.087	**0.024**	0.999
Week 16	39.65 ± 0.08 [39.50, 39.81]	40.11 ± 0.08 [39.95, 40.27]	40.18 ± 0.08 [40.02, 40.35]	**0.003**	< 0.001	0.999
Δ Week 8 (change from baseline)	-0.37 ± 0.10 [-0.57, -0.18]	-0.04 ± 0.10 [-0.24, 0.16]	0.01 ± 0.10 [-0.19, 0.22]	0.193	0.080	0.999
Δ Week 16 (change from baseline)	-0.24 ± 0.10 [-0.43, -0.04]	0.21 ± 0.10 [0.01, 0.41]	0.28 ± 0.10 [0.08, 0.49]	**0.021**	**0.004**	0.997
(f) SMM (kg)						
Baseline (Week 0)	21.39 ± 0.04 [21.31, 21.48]	21.39 ± 0.05 [21.31, 21.48]	21.40 ± 0.05 [21.31, 21.49]	0.999	0.999	0.999
Week 8	21.20 ± 0.04 [21.11, 21.28]	21.39 ± 0.05 [21.30, 21.48]	21.43 ± 0.05 [21.34, 21.52]	0.052	**0.007**	0.999
Week 16	21.31 ± 0.04 [21.22, 21.39]	21.55 ± 0.05 [21.46, 21.64]	21.61 ± 0.05 [21.52, 21.70]	**0.004**	< 0.001	0.994
Δ Week 8 (change from baseline)	-0.19 ± 0.05 [-0.30, -0.09]	0.00 ± 0.06 [-0.11, 0.11]	0.04 ± 0.06 [-0.07, 0.15]	0.138	**0.034**	0.994
Δ Week 16 (change from baseline)	-0.08 ± 0.05 [-0.19, 0.02]	0.16 ± 0.06 [0.05, 0.27]	0.21 ± 0.06 [0.10, 0.32]	**0.026**	**0.002**	0.979
(g) SMI (kg/m²)						
Baseline (Week 0)	6.29 ± 0.01 [6.26, 6.32]	6.29 ± 0.01 [6.26, 6.32]	6.29 ± 0.01 [6.26, 6.32]	0.999	0.999	0.999
Week 8	6.20 ± 0.01 [6.18, 6.23]	6.27 ± 0.01 [6.24, 6.30]	6.27 ± 0.01 [6.24, 6.30]	**0.049**	0.056	0.999
Week 16	6.20 ± 0.01 [6.17, 6.22]	6.27 ± 0.01 [6.25, 6.30]	6.28 ± 0.01 [6.25, 6.31]	**0.004**	**0.001**	0.999
Δ Week 8 (change from baseline)	-0.09 ± 0.02 [-0.12, -0.05]	-0.02 ± 0.02 [-0.06, 0.01]	-0.02 ± 0.02 [-0.06, 0.01]	0.116	0.137	0.999
Δ Week 16 (change from baseline)	-0.09 ± 0.02 [-0.13, -0.06]	-0.01 ± 0.02 [-0.05, 0.02]	-0.01 ± 0.02 [-0.05, 0.03]	**0.024**	**0.016**	0.999
(h) ECW/TBW (ratio)						
Baseline (Week 0)	0.390 ± 0.000 [0.390, 0.391]	0.390 ± 0.000 [0.389, 0.390]	0.390 ± 0.000 [0.389, 0.390]	0.999	0.999	0.999
Week 8	0.389 ± 0.000 [0.388, 0.390]	0.389 ± 0.000 [0.388, 0.390]	0.389 ± 0.000 [0.388, 0.389]	0.999	0.999	0.999
Week 16	0.388 ± 0.000 [0.387, 0.388]	0.389 ± 0.000 [0.388, 0.389]	0.388 ± 0.000 [0.388, 0.389]	0.329	0.998	0.828
Δ Week 8 (change from baseline)	−0.0008 ± 0.0004 [− 0.0016, 0.0000]	−0.0006 ± 0.0004 [− 0.0014, 0.0003]	−0.0001 ± 0.0004 [− 0.0010, 0.0007]	0.999	0.891	0.983
Δ Week 16 (change from baseline)	−0.0022 ± 0.0004 [− 0.0031, − 0.0014]	−0.0010 ± 0.0004 [− 0.0018, − 0.0001]	−0.0011 ± 0.0004 [− 0.0020, − 0.0002]	0.316	0.413	0.999

*Body weight, percent body fat, BMI, SLM, FFM, SMM, SMI, and ECW/TBW are shown for the NI, HC, and HC+MILK groups at baseline, week 8, and week 16. Values are least-squares means ± SE with 95% confidence intervals. Δ Week 8 and Δ Week 16 indicate changes from baseline. Tukey-adjusted *P* values are shown for pairwise comparisons among the six time-by-group estimated values. *P* values reported as 0.000 in the original output are presented as < 0.001

*BMI* body mass index, *SLM* soft lean mass, *FFM* fat-free mass, *SMM* skeletal muscle mass, *SMI* skeletal muscle index, *ECW/TBW* extracellular water/total body water, *NI* no intake, *HC* high cacao, *HC+MILK* high cacao plus milk-protein beverage

Bold values indicate statistically significant Tukey-adjusted *P* values (*P* < 0.05)

At week 16, body-weight changes were + 0.17, + 0.54, and + 0.78 kg in NI, HC, and HC+MILK, respectively. The contrasts were HC − NI + 0.38 kg (95% CI − 0.13 to 0.88; *P* = 0.277), HC+MILK − NI + 0.61 kg (0.10 to 1.12; *P* = 0.009), and HC+MILK − HC + 0.23 kg (− 0.28 to 0.75; *P* = 0.784). BMI changes were + 0.08, + 0.21, and + 0.31 kg/m², respectively; the contrasts were + 0.13 kg/m² (− 0.07 to 0.32; *P* = 0.444), + 0.23 kg/m² (0.03 to 0.43; *P* = 0.011), and + 0.11 kg/m² (− 0.09 to 0.31; *P* = 0.645), respectively (Table 3). Percent body fat and ECW/TBW showed no significant differential changes among groups.

**Fig. 2:**
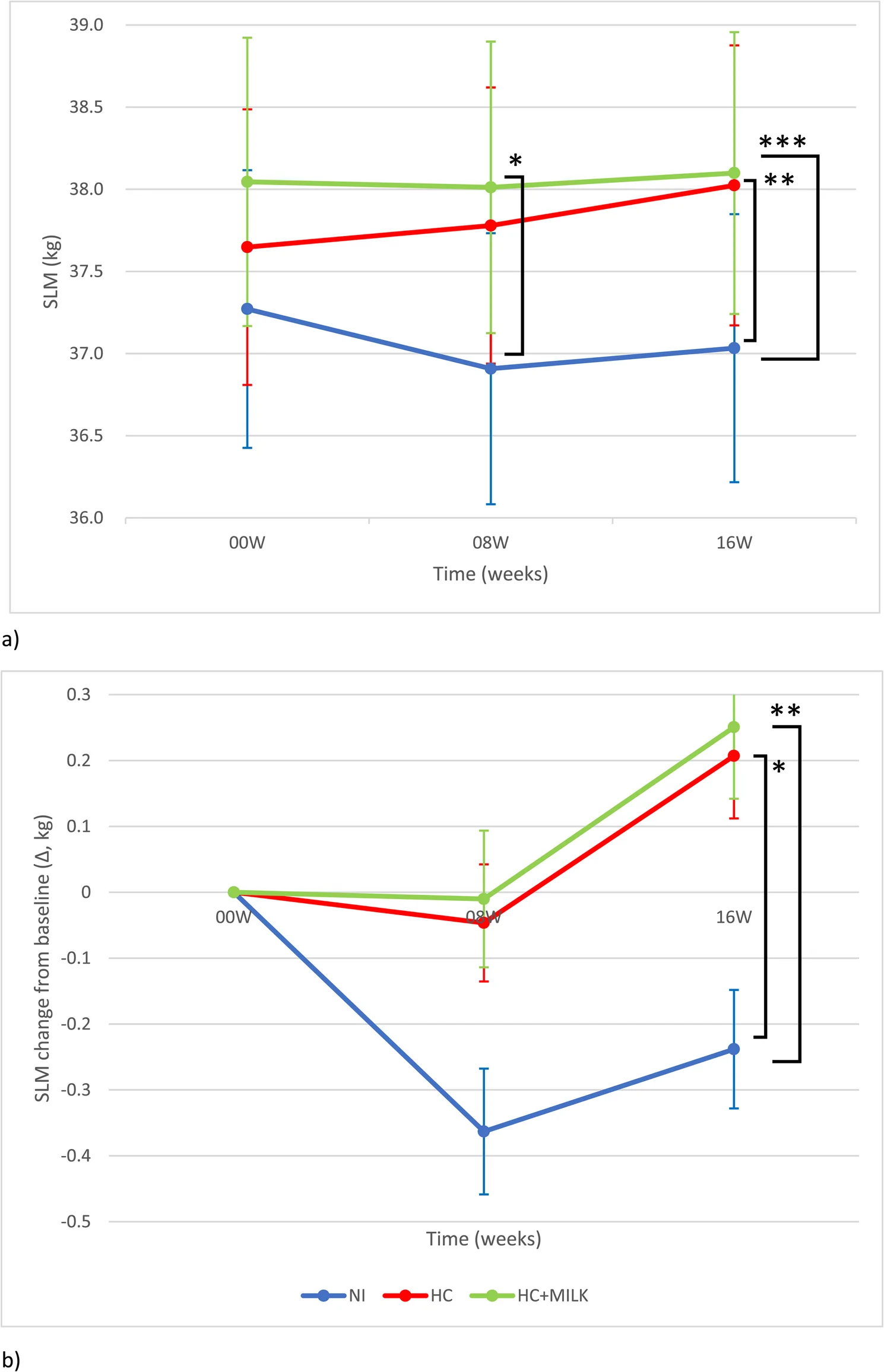
Changes in soft lean mass (SLM). **a** Observed values (mean ± SD) at baseline, week 8, and week 16 for the NI, HC, and HC+MILK groups. **b** Changes from baseline (Δ; mean ± SD) at weeks 8 and 16. Statistical inference was based on longitudinal linear mixed models with group, time, and group×time effects and a participant-specific random intercept; observed-value models also included the corresponding baseline value. Pairwise comparisons were adjusted using Tukey’s method. Week 16 was the prespecified primary assessment. Analyses were performed in the PPS. Significance after Tukey adjustment is indicated (*P < 0.05, **P < 0.01, ***P < 0.001). SD, standard deviation; NI, no intake; HC, high-cacao chocolate; HC+MILK, high-cacao chocolate plus milk-protein beverage; PPS, per-protocol set

Figure 2 shows the SLM trajectories, Additional file 1 shows body-weight trajectories, and Table 3 summarizes observed values and changes for all eight body-composition outcomes. Tukey-adjusted week-16 between-group differences in change and simultaneous 95% confidence intervals are reported in the text, and Additional file 6 provides baseline-adjusted standardized mean differences.

Physical-function outcomes, clinical and biochemical outcomes, step counts, and dietary intake are summarized in Additional files 2–5.

### Timepoint-specific findings

At week 8, observed-value comparisons favored HC+MILK over NI for body weight, SLM, FFM, and SMM, whereas the change-from-baseline comparison was significant only for SMM. By week 16, changes in all four muscle-related outcomes differed significantly between each HC-containing group and NI, while no clear difference was detected between HC and HC+MILK.

### Secondary outcomes

No significant time-by-group interactions were observed for the 4-m walk, five-times sit-to-stand test, ZaRits power, speed, or balance, handgrip strength, step counts, vital signs, inflammatory or oxidative-stress markers, lipid variables, or glucose levels. For step counts, time-by-group P values were 0.818 for observed values and 0.741 for changes from baseline (Additional file 4). Several physical-function measures changed over time across all groups. Physical-function and clinical/biochemical outcomes are summarized in Additional files 2 and 3.

### Dietary intake

Estimated dietary energy and protein intakes showed no significant time-by-group interactions (P = 0.387 and P = 0.156, respectively). Mean estimated protein intake was 1.25, 1.34, and 1.23 g/kg/day at baseline and 1.32, 1.31, and 1.22 g/kg/day at week 16 in NI, HC, and HC+MILK, respectively. Corresponding estimated energy intake was 29.8, 31.3, and 30.2 kcal/kg/day at baseline and 30.8, 30.8, and 30.1 kcal/kg/day at week 16 (Additional file 5).

### Adverse events

Three SAEs occurred but were unrelated to the study products. No product-related AEs or SAEs were observed, and no clinically relevant safety concerns were identified.

## Discussion

In this exploratory 16-week randomized trial, HC and HC+MILK showed statistically significant favorable changes versus NI in the representative BIA-derived outcomes SLM and SMI. FFM and SMM, which were derived from the same BIA assessment and are therefore closely related rather than independent outcomes, showed directionally consistent findings. At week 16, the estimated differences versus NI were approximately 0.45–0.49 kg for SLM and 0.08 kg/m² for SMI, with supportive differences of 0.45–0.52 kg for FFM and 0.24–0.30 kg for SMM; baseline-adjusted standardized mean differences were generally approximately 0.5–0.6. SLM, FFM, and SMM increased in the HC-containing groups while declining in NI, and SMI was largely maintained in the HC-containing groups while declining in NI. This pattern supports a potential benefit for maintaining or increasing BIA-estimated muscle mass under habitual activity conditions. In contrast, the HC+MILK − HC differences were close to zero, indicating no clear incremental benefit from adding MILK under the present conditions. Although the between-group differences were statistically significant, no clinically meaningful threshold was prespecified, established minimal clinically important differences for these BIA-derived outcomes are unavailable, and no intervention-specific functional improvement was detected. Their clinical relevance therefore remains uncertain.

The HC used in this trial contained cacao-derived flavanols and procyanidins. These compounds have been proposed to influence muscle-related outcomes through redox-related pathways, vascular or perfusion-related actions, and modulation of low-grade inflammation [13, 16, 17]. Age-related disruption of redox homeostasis in skeletal muscle is associated with sarcopenia [26], and bidirectional relationships exist between sarcopenia and metabolic dysregulation [27]. However, neither circulating 8-OHdG nor hs-CRP showed a significant time-by-group interaction in the present study, and no differential changes were observed in lipid variables or glucose levels. The data therefore do not support a specific systemic oxidative-stress, inflammatory, glycemic, or lipid mechanism. Future studies should incorporate vascular-function assessments, more direct mechanistic biomarkers, and prospectively defined analyses according to baseline inflammatory status.

Milk-protein supplementation, particularly when combined with exercise, has been reported to increase muscle mass in older adults [12]. Nevertheless, the HC+MILK group did not differ clearly from HC for the muscle-related outcomes. Habitual protein intake at baseline was already approximately 1.2–1.3 g/kg/day across groups, which may have limited the opportunity to detect an additional benefit from MILK. Moreover, estimated protein intake at week 16 did not increase by the nominal 14.8 g/day supplied by MILK, potentially reflecting incomplete capture of the study product by the BDHQ, displacement of other protein-containing foods, self-report error, or a combination of these factors.

Energy balance and food displacement provide alternative context for the findings. The assigned study products provided 85 kcal/day in HC and 189 kcal/day in HC+MILK; however, these nominal values cannot be assumed to represent equivalent net increases in total energy intake because comprehensive food records were not collected. The additional energy may have partially offset an energy deficit in some participants, whereas study foods may also have displaced habitual foods. Estimated energy intake per kilogram did not show a differential change among groups. Because an isocaloric placebo was not used, the contributions of energy intake, food displacement, and expectation effects cannot be separated from other intervention effects.

The trial was conducted from summer to winter. Seasonal changes in body composition, metabolism, and physical activity have been reported in older populations and snowy regions [28–32]. In this trial, step counts declined over time in all groups, with no significant time-by-group interaction. Thus, a preferential increase in ambulatory activity in the intervention groups does not appear to explain the observed pattern. Nevertheless, step counts do not capture resistance exercise or all forms of mechanical loading, and changes in exercise type, intensity, and duration were not assessed repeatedly. The decline observed in NI is directionally compatible with age-related loss of muscle mass, but a 16-week BIA change should not be equated with an annual age-related trajectory because seasonality, hydration, measurement variability, and activity changes may also contribute. Within these constraints, the observed pattern indicates maintenance or increases in BIA-estimated muscle-related indices in the HC-containing groups under usual daily-life activity; it does not by itself establish anatomical muscle hypertrophy or prevention of sarcopenia.

The absence of a prescribed structured exercise program was intentional because the trial was designed to evaluate the nutritional interventions under habitual lifestyle conditions. Physical-function outcomes were prespecified to determine whether body-composition changes were accompanied by functional changes, but no intervention-specific effects were detected. Several measures changed over time in all groups. These common temporal changes may reflect genuine physiological change, familiarization or practice effects from repeated testing, or both. Because no separate familiarization session was included, these possibilities cannot be distinguished, and practice effects may have reduced sensitivity to small between-group differences. Longer studies designed around functional endpoints are needed.

Body weight and BMI increased more in HC+MILK than in NI, whereas percent body fat did not show a differential change. Together with the BIA-derived SLM, FFM, and SMM findings and the absence of a group difference in ECW/TBW, this pattern is consistent with at least part of the weight change reflecting BIA-estimated fat-free and muscle-related components rather than fat accumulation or extracellular-water imbalance alone. The increases in BIA-estimated SLM, FFM, and SMM are therefore valid findings within the measurement framework used in this trial. However, BIA cannot determine tissue composition with the precision of DXA or directly confirm anatomical muscle hypertrophy, and hydration, measurement variability, energy balance, and food displacement may have contributed.

The strengths of this trial include its prespecified randomized design, high adherence, repeated assessments, and transparent reporting of multiple body-composition indices.

The study also has important limitations. It was exploratory and open-label, without a placebo or isocaloric control, and the eight primary indices were correlated outputs from the same BIA device rather than independent measures. DXA confirmation and bone-mineral assessment were not available. The BDHQ was self-reported and intended primarily to characterize habitual intake; comprehensive food records and 24-h recalls were not collected, limiting assessment of day-to-day variation, study-food capture, and displacement of habitual foods. Residual habitual cacao intake below the exclusion threshold was not quantified. Baseline nutritional and functional status was heterogeneous, spanning participants classified as robust, prefrail, or frail according to the 25-item frailty checklist, which may have increased response variability. Post hoc efficacy analyses by frailty or low-muscle-mass status were not performed because they were not prespecified and the resulting subgroups were limited in size. Although general exercise habits were assessed at baseline and step counts were measured at three timepoints, resistance exercise and changes in exercise type, intensity, and duration were not assessed comprehensively. The 3-day step-count periods did not capture all activity. The 16-week duration was insufficient to evaluate durability or clinical outcomes, and the regional and seasonal setting limits generalizability. These limitations do not negate the observed BIA-derived between-group differences, but they limit conclusions about anatomical hypertrophy and clinical importance. Future confirmatory studies should use prospectively selected primary outcomes, adequate power, longer follow-up, isocaloric placebo controls, DXA or other reference methods, detailed dietary and exercise assessment, and functional and clinical endpoints.

## Conclusions

In community-dwelling older adults, 16 weeks of daily HC or HC+MILK showed statistically significant favorable between-group differences in BIA-derived muscle-related indices compared with NI. SLM, FFM, and SMM increased in the HC-containing groups while declining in NI, and SMI was largely maintained, supporting a potential benefit for maintaining or increasing BIA-estimated muscle mass under habitual activity conditions. No clear incremental benefit of adding MILK to HC was detected, and no intervention-specific physical-function effect was observed. The use of BIA and the absence of functional improvement mean that confirmation of anatomical hypertrophy and the clinical relevance of the observed differences require further study. Confirmatory trials using isocaloric controls, reference body-composition methods, detailed exercise assessment, and longer follow-up with functional and clinical outcomes are required.

## Supplementary Information

Additional file 1: Supplementary Figure S1. Changes in body weight.

Additional file 2: Table S1. Physical-function outcomes at baseline, week 8, and week 16.

Additional file 3: Table S2. Clinical and biochemical outcomes at baseline, week 8, and week 16.

Additional file 4: Table S3. Step counts (steps/day) at baseline, week 8, and week 16.

Additional file 5: Table S4. Dietary intake at baseline and week 16 estimated using the BDHQ.

Additional file 6: Table S5. Baseline-adjusted standardized mean differences for body-composition outcomes at week 8 and week 16.

## Data Availability

Upon acceptance, we will deposit fully anonymized individual-level data, a data dictionary, and reproducible R scripts for the tables and figures in Dryad under CC0 (DOI will be added after acceptance). During peer review, editors and reviewers will receive a private anonymized access link. The specifications of the questionnaires, derived variables, and key model settings are summarized in the Additional Files. The SPIRIT-conformant protocol and statistical analysis plan (SAP) will be provided to the journal during review and after publication upon reasonable request.
