# Supplementary material for: Effects of daily intake of cacao polyphenols, alone or with milk protein, on skeletal muscle mass in community-dwelling older adults: an exploratory 16-week randomized, open-label, parallel-group trial
Source: BMC Nutr. 2026 Aug 3;12:229. doi: 10.1186/s40795-026-01445-7 (PMC13721333; doi:10.1186/s40795-026-01445-7)
Supplement: Supplementary file 1 [file 40795_2026_1445_MOESM1_ESM.docx]

**

*

Weight (kg)

| Time (weeks) | NI | HC | HC+MILK |
| --- | --- | --- | --- |
| 00W | 55.0382 | 54.2528 | 55.1845 |
| 08W | 55.0737 | 54.4958 | 55.4929 |
| 16W | 55.2039 | 54.7944 | 55.6986 |

a)

*

Weight change from baseline (Δ, kg)

| Time (weeks) | NI | HC | HC+MILK |
| --- | --- | --- | --- |
| 00W | 0 | 0 | 0 |
| 08W | 0.0355263 | 0.243056 | 0.49 |
| 16W | 0.165789 | 0.541667 | 0.775362 |

b)
