# Supplementary material for: Effects of daily intake of cacao polyphenols, alone or with milk protein, on skeletal muscle mass in community-dwelling older adults: an exploratory 16-week randomized, open-label, parallel-group trial
Source: BMC Nutr. 2026 Aug 3;12:229. doi: 10.1186/s40795-026-01445-7 (PMC13721333; doi:10.1186/s40795-026-01445-7)
Supplement: Supplementary file 2 [file 40795_2026_1445_MOESM2_ESM.docx]

| **Table S1. Physical-function outcomes at baseline, week 8, and week 16** | |  |  |  |  |  |  |
| --- | --- | --- | --- | --- | --- | --- | --- |
| **Time** | **NI n=76** | **HC n=72** | **HC+MILK n=71** | **P-value** | | | |
|  |  |  |  |  | Time | Group | Time×Group |
| **(a) 4-m walking time (s)** |  |  |  |  |  |  |  |
| Baseline (0W) | 3.12 ± 0.03 [3.06, 3.17] | 3.12 ± 0.03 [3.06, 3.17] | 3.11 ± 0.03 [3.05, 3.16] | Observed values | <0.001 | 0.114 | 0.388 |
| Week 8 | 3.01 ± 0.03 [2.96, 3.06] | 2.93 ± 0.03 [2.88, 2.98] | 2.94 ± 0.03 [2.89, 3.00] |  |  |  |  |
| Week 16 | 3.08 ± 0.03 [3.02, 3.13] | 3.00 ± 0.03 [2.95, 3.05] | 3.03 ± 0.03 [2.98, 3.09] |  |  |  |  |
| Δ Week 8 (change from baseline) | -0.11 ± 0.03 [-0.17, -0.04] | -0.19 ± 0.04 [-0.26, -0.12] | -0.17 ± 0.04 [-0.23, -0.10] | Changes from baseline | <0.001 | 0.173 | 0.830 |
| Δ Week 16 (change from baseline) | -0.04 ± 0.03 [-0.11, 0.03] | -0.12 ± 0.04 [-0.18, -0.05] | -0.07 ± 0.04 [-0.14, -0.00] |  |  |  |  |
| **(b) Five-times sit-to-stand time (s)** |  |  |  |  |  |  |  |
| Baseline (0W) | 6.73 ± 0.12 [6.51, 6.96] | 6.88 ± 0.12 [6.65, 7.11] | 6.84 ± 0.12 [6.60, 7.07] | Observed values | <0.001 | 0.313 | 0.091 |
| Week 8 | 6.82 ± 0.12 [6.60, 7.05] | 6.74 ± 0.12 [6.51, 6.98] | 6.45 ± 0.12 [6.21, 6.69] |  |  |  |  |
| Week 16 | 6.44 ± 0.12 [6.21, 6.66] | 6.14 ± 0.12 [5.90, 6.37] | 6.20 ± 0.12 [5.96, 6.44] |  |  |  |  |
| Δ Week 8 (change from baseline) | 0.09 ± 0.15 [-0.21, 0.39] | -0.14 ± 0.16 [-0.44, 0.17] | -0.39 ± 0.16 [-0.70, -0.08] | Changes from baseline | <0.001 | 0.067 | 0.277 |
| Δ Week 16 (change from baseline) | -0.30 ± 0.15 [-0.60, -0.00] | -0.74 ± 0.16 [-1.05, -0.44] | -0.64 ± 0.16 [-0.95, -0.33] |  |  |  |  |
| **(c) ZaRits Power (F/w)** |  |  |  |  |  |  |  |
| Baseline (0W) | 1.30 ± 0.01 [1.29, 1.31] | 1.30 ± 0.01 [1.29, 1.32] | 1.30 ± 0.01 [1.29, 1.32] | Observed values | <0.001 | 0.599 | 0.742 |
| Week 8 | 1.32 ± 0.01 [1.30, 1.33] | 1.32 ± 0.01 [1.31, 1.33] | 1.33 ± 0.01 [1.32, 1.34] |  |  |  |  |
| Week 16 | 1.33 ± 0.01 [1.32, 1.34] | 1.32 ± 0.01 [1.31, 1.33] | 1.33 ± 0.01 [1.32, 1.34] |  |  |  |  |
| Δ Week 8 (change from baseline) | 0.02 ± 0.01 [0.00, 0.03] | 0.02 ± 0.01 [0.00, 0.03] | 0.03 ± 0.01 [0.01, 0.04] | Changes from baseline | 0.223 | 0.737 | 0.474 |
| Δ Week 16 (change from baseline) | 0.03 ± 0.01 [0.01, 0.04] | 0.02 ± 0.01 [0.00, 0.03] | 0.03 ± 0.01 [0.01, 0.04] |  |  |  |  |
| **(d) ZaRits Speed (RFD/w)** |  |  |  |  |  |  |  |
| Baseline (0W) | 9.00 ± 0.12 [8.75, 9.24] | 9.11 ± 0.13 [8.86, 9.36] | 9.12 ± 0.13 [8.87, 9.38] | Observed values | <0.001 | 0.765 | 0.102 |
| Week 8 | 9.27 ± 0.12 [9.02, 9.51] | 9.36 ± 0.13 [9.11, 9.61] | 9.45 ± 0.13 [9.19, 9.70] |  |  |  |  |
| Week 16 | 10.03 ± 0.12 [9.79, 10.27] | 9.61 ± 0.13 [9.36, 9.86] | 9.75 ± 0.13 [9.49, 10.00] |  |  |  |  |
| Δ Week 8 (change from baseline) | 0.27 ± 0.16 [-0.05, 0.59] | 0.25 ± 0.17 [-0.08, 0.58] | 0.32 ± 0.17 [-0.01, 0.65] | Changes from baseline | <0.001 | 0.377 | 0.062 |
| Δ Week 16 (change from baseline) | 1.03 ± 0.16 [0.72, 1.35] | 0.50 ± 0.17 [0.18, 0.83] | 0.63 ± 0.17 [0.29, 0.96] |  |  |  |  |
| **(e) ZaRits Balance (points)** |  |  |  |  |  |  |  |
| Baseline (0W) | 51.91 ± 0.70 [50.54, 53.27] | 52.47 ± 0.72 [51.06, 53.87] | 51.70 ± 0.72 [50.28, 53.12] | Observed values | 0.003 | 0.103 | 0.454 |
| Week 8 | 51.96 ± 0.70 [50.59, 53.33] | 52.70 ± 0.72 [51.29, 54.11] | 53.16 ± 0.73 [51.73, 54.59] |  |  |  |  |
| Week 16 | 52.64 ± 0.70 [51.27, 54.01] | 54.20 ± 0.72 [52.79, 55.61] | 55.10 ± 0.73 [53.66, 56.54] |  |  |  |  |
| Δ Week 8 (change from baseline) | 0.05 ± 1.00 [-1.91, 2.01] | 0.24 ± 1.02 [-1.78, 2.25] | 1.48 ± 1.04 [-0.56, 3.51] | Changes from baseline | 0.019 | 0.242 | 0.666 |
| Δ Week 16 (change from baseline) | 0.74 ± 1.00 [-1.22, 2.69] | 1.74 ± 1.02 [-0.28, 3.75] | 3.43 ± 1.04 [1.38, 5.48] |  |  |  |  |
| **(f) Handgrip strength (kg)** |  |  |  |  |  |  |  |
| Baseline (0W) | 25.10 ± 0.18 [24.75, 25.44] | 25.11 ± 0.18 [24.76, 25.47] | 25.12 ± 0.18 [24.76, 25.47] | Observed values | <0.001 | 0.269 | 0.627 |
| Week 8 | 25.48 ± 0.18 [25.14, 25.83] | 25.73 ± 0.18 [25.38, 26.08] | 25.89 ± 0.18 [25.53, 26.24] |  |  |  |  |
| Week 16 | 25.04 ± 0.18 [24.69, 25.39] | 25.32 ± 0.18 [24.96, 25.67] | 25.50 ± 0.18 [25.14, 25.86] |  |  |  |  |
| Δ Week 8 (change from baseline) | 0.39 ± 0.22 [-0.04, 0.82] | 0.62 ± 0.23 [0.17, 1.06] | 0.78 ± 0.23 [0.33, 1.22] | Changes from baseline | <0.001 | 0.338 | 0.975 |
| Δ Week 16 (change from baseline) | -0.05 ± 0.22 [-0.48, 0.38] | 0.21 ± 0.23 [-0.24, 0.65] | 0.40 ± 0.23 [-0.05, 0.85] |  |  |  |  |
| **Legend:** 4-m walking time, five-times sit-to-stand time, ZaRits power, ZaRits speed, ZaRits balance, and handgrip strength for the NI, HC, and HC+MILK groups. Values are LS mean ± SE with 95% confidence intervals in brackets. P-values are from linear mixed models with fixed effects for group, time, and group×time. Tukey-adjusted post-hoc comparisons were not performed because the group×time interaction was not significant. NI, no intake; HC, high-cacao chocolate; HC+MILK, high-cacao chocolate plus milk-protein beverage. | | | | | | | |
