# Supplementary material for: Effects of daily intake of cacao polyphenols, alone or with milk protein, on skeletal muscle mass in community-dwelling older adults: an exploratory 16-week randomized, open-label, parallel-group trial
Source: BMC Nutr. 2026 Aug 3;12:229. doi: 10.1186/s40795-026-01445-7 (PMC13721333; doi:10.1186/s40795-026-01445-7)
Supplement: Supplementary file 3 [file 40795_2026_1445_MOESM3_ESM.docx]

| **Table S2. Clinical and biochemical outcomes at baseline, week 8, and week 16.** | | |  |  |  |  |  |
| --- | --- | --- | --- | --- | --- | --- | --- |
| **Time** | **NI n=76** | **HC n=72** | **HC+MILK n=71** | **P-value** | | | |
|  |  |  |  |  | Time | Group | Time×Group |
| **(a) Clinic SBP (mmHg)** |  |  |  |  |  |  |  |
| Baseline (0W) | 133.27 ± 1.12 [131.06, 135.47] | 134.08 ± 1.14 [131.84, 136.33] | 134.21 ± 1.15 [131.95, 136.48] | Observed values | 0.202 | 0.962 | 0.688 |
| Week 8 | 133.16 ± 1.12 [130.95, 135.37] | 132.63 ± 1.14 [130.38, 134.87] | 131.25 ± 1.16 [128.97, 133.53] |  |  |  |  |
| Week 16 | 133.07 ± 1.12 [130.86, 135.27] | 132.86 ± 1.14 [130.61, 135.11] | 133.29 ± 1.17 [130.99, 135.58] |  |  |  |  |
| Δ Week 8 (change from baseline) | -0.11 ± 1.47 [-2.99, 2.78] | -1.46 ± 1.50 [-4.40, 1.49] | -2.96 ± 1.52 [-5.94, 0.02] | Changes from baseline | 0.355 | 0.628 | 0.496 |
| Δ Week 16 (change from baseline) | -0.20 ± 1.47 [-3.09, 2.69] | -1.22 ± 1.50 [-4.17, 1.72] | -0.86 ± 1.52 [-3.85, 2.14] |  |  |  |  |
| **(b) Clinic DBP (mmHg)** |  |  |  |  |  |  |  |
| Baseline (0W) | 85.15 ± 0.63 [83.91, 86.39] | 85.41 ± 0.64 [84.14, 86.68] | 85.33 ± 0.65 [84.05, 86.60] | Observed values | 0.484 | 0.879 | 0.838 |
| Week 8 | 84.82 ± 0.63 [83.58, 86.06] | 85.38 ± 0.64 [84.12, 86.65] | 85.00 ± 0.65 [83.72, 86.28] |  |  |  |  |
| Week 16 | 86.09 ± 0.63 [84.85, 87.33] | 85.65 ± 0.64 [84.38, 86.91] | 85.16 ± 0.66 [83.87, 86.46] |  |  |  |  |
| Δ Week 8 (change from baseline) | -0.33 ± 0.81 [-1.92, 1.25] | -0.03 ± 0.82 [-1.65, 1.59] | -0.32 ± 0.83 [-1.96, 1.32] | Changes from baseline | 0.229 | 0.864 | 0.559 |
| Δ Week 16 (change from baseline) | 0.93 ± 0.81 [-0.65, 2.52] | 0.24 ± 0.82 [-1.38, 1.85] | -0.16 ± 0.84 [-1.81, 1.48] |  |  |  |  |
| **(c) Serum 8-hydroxy-2'-deoxyguanosine (8-OHdG), ng/mL** |  |  |  |  |  |  |  |
| Baseline (0W) | 0.20 ± 0.03 [0.14, 0.27] | 0.20 ± 0.03 [0.13, 0.26] | 0.17 ± 0.03 [0.10, 0.24] | Observed values | <0.001 | 0.229 | 0.485 |
| Week 8 | 0.33 ± 0.03 [0.27, 0.40] | 0.30 ± 0.03 [0.24, 0.37] | 0.35 ± 0.03 [0.28, 0.41] |  |  |  |  |
| Week 16 | 0.29 ± 0.03 [0.23, 0.35] | 0.19 ± 0.03 [0.12, 0.25] | 0.24 ± 0.03 [0.17, 0.30] |  |  |  |  |
| Δ Week 8 (change from baseline) | 0.13 ± 0.04 [0.04, 0.22] | 0.10 ± 0.05 [0.01, 0.19] | 0.18 ± 0.05 [0.09, 0.27] | Changes from baseline | 0.004 | 0.305 | 0.571 |
| Δ Week 16 (change from baseline) | 0.09 ± 0.04 [0.00, 0.17] | -0.01 ± 0.05 [-0.10, 0.08] | 0.07 ± 0.05 [-0.03, 0.16] |  |  |  |  |
| **(d) hs-CRP (mg/dL)** |  |  |  |  |  |  |  |
| Baseline (0W) | 0.08 ± 0.03 [0.03, 0.14] | 0.09 ± 0.03 [0.03, 0.15] | 0.08 ± 0.03 [0.02, 0.14] | Observed values | 0.577 | 0.984 | 0.276 |
| Week 8 | 0.06 ± 0.03 [0.00, 0.12] | 0.13 ± 0.03 [0.07, 0.19] | 0.11 ± 0.03 [0.05, 0.17] |  |  |  |  |
| Week 16 | 0.15 ± 0.03 [0.09, 0.21] | 0.08 ± 0.03 [0.03, 0.14] | 0.10 ± 0.03 [0.04, 0.16] |  |  |  |  |
| Δ Week 8 (change from baseline) | -0.02 ± 0.04 [-0.10, 0.05] | 0.04 ± 0.04 [-0.04, 0.12] | 0.02 ± 0.04 [-0.06, 0.10] | Changes from baseline | 0.683 | 0.998 | 0.132 |
| Δ Week 16 (change from baseline) | 0.06 ± 0.04 [-0.01, 0.14] | 0.00 ± 0.04 [-0.08, 0.07] | 0.02 ± 0.04 [-0.06, 0.10] |  |  |  |  |
| **(e) TC (mg/dL)** |  |  |  |  |  |  |  |
| Baseline (0W) | 230.09 ± 1.99 [226.17, 234.00] | 230.22 ± 2.05 [226.20, 234.24] | 230.08 ± 2.06 [226.03, 234.13] | Observed values | 0.001 | 0.884 | 0.880 |
| Week 8 | 234.07 ± 1.99 [230.16, 237.99] | 235.67 ± 2.05 [231.64, 239.69] | 234.67 ± 2.08 [230.60, 238.75] |  |  |  |  |
| Week 16 | 236.00 ± 1.99 [232.08, 239.91] | 235.05 ± 2.05 [231.03, 239.08] | 233.23 ± 2.09 [229.13, 237.34] |  |  |  |  |
| Δ Week 8 (change from baseline) | 3.99 ± 2.48 [-0.90, 8.87] | 5.44 ± 2.55 [0.42, 10.47] | 4.64 ± 2.58 [-0.44, 9.72] | Changes from baseline | 0.961 | 0.914 | 0.580 |
| Δ Week 16 (change from baseline) | 5.91 ± 2.48 [1.02, 10.8] | 4.83 ± 2.55 [-0.19, 9.85] | 3.12 ± 2.60 [-1.99, 8.23] |  |  |  |  |
| **(f) LDL-C (mg/dL)** |  |  |  |  |  |  |  |
| Baseline (0W) | 133.29 ± 1.74 [129.87, 136.72] | 133.31 ± 1.79 [129.79, 136.83] | 133.63 ± 1.80 [130.09, 137.18] | Observed values | 0.056 | 0.789 | 0.875 |
| Week 8 | 136.81 ± 1.74 [133.38, 140.23] | 135.99 ± 1.79 [132.47, 139.51] | 136.40 ± 1.82 [132.84, 139.97] |  |  |  |  |
| Week 16 | 136.99 ± 1.74 [133.57, 140.42] | 134.25 ± 1.79 [130.74, 137.77] | 134.46 ± 1.83 [130.87, 138.05] |  |  |  |  |
| Δ Week 8 (change from baseline) | 3.51 ± 2.18 [-0.77, 7.80] | 2.68 ± 2.24 [-1.72, 7.08] | 2.80 ± 2.26 [-1.65, 7.26] | Changes from baseline | 0.311 | 0.751 | 0.700 |
| Δ Week 16 (change from baseline) | 3.70 ± 2.18 [-0.59, 7.98] | 0.94 ± 2.24 [-3.46, 5.35] | 0.74 ± 2.28 [-3.74, 5.21] |  |  |  |  |
| **(g) HDL-C (mg/dL)** |  |  |  |  |  |  |  |
| Baseline (0W) | 73.91 ± 0.81 [72.31, 75.51] | 73.91 ± 0.84 [72.27, 75.55] | 73.89 ± 0.84 [72.24, 75.54] | Observed values | <0.001 | 0.023 | 0.117 |
| Week 8 | 75.59 ± 0.81 [74.00, 77.19] | 78.85 ± 0.84 [77.21, 80.49] | 76.74 ± 0.85 [75.08, 78.41] |  |  |  |  |
| Week 16 | 77.15 ± 0.81 [75.55, 78.74] | 80.44 ± 0.84 [78.80, 82.08] | 77.74 ± 0.85 [76.07, 79.42] |  |  |  |  |
| Δ Week 8 (change from baseline) | 1.68 ± 1.00 [-0.27, 3.64] | 4.94 ± 1.02 [2.93, 6.96] | 2.88 ± 1.04 [0.84, 4.92] | Changes from baseline | 0.027 | 0.023 | 0.906 |
| Δ Week 16 (change from baseline) | 3.24 ± 1.00 [1.28, 5.19] | 6.53 ± 1.02 [4.52, 8.54] | 3.86 ± 1.04 [1.81, 5.91] |  |  |  |  |
| **(h) TG (mg/dL)** |  |  |  |  |  |  |  |
| Baseline (0W) | 99.82 ± 4.90 [90.20, 109.44] | 100.34 ± 5.03 [90.45, 110.22] | 98.95 ± 5.07 [88.99, 108.90] | Observed values | 0.268 | 0.586 | 0.537 |
| Week 8 | 103.07 ± 4.90 [93.45, 112.69] | 89.75 ± 5.03 [79.87, 99.64] | 96.53 ± 5.10 [86.51, 106.55] |  |  |  |  |
| Week 16 | 94.92 ± 4.90 [85.30, 104.54] | 91.94 ± 5.03 [82.05, 101.82] | 95.48 ± 5.13 [85.40, 105.56] |  |  |  |  |
| Δ Week 8 (change from baseline) | 3.25 ± 6.21 [-8.97, 15.47] | -10.58 ± 6.38 [-23.14, 1.97] | -2.51 ± 6.45 [-15.20, 10.19] | Changes from baseline | 0.480 | 0.538 | 0.330 |
| Δ Week 16 (change from baseline) | -4.89 ± 6.21 [-17.12, 7.33] | -8.40 ± 6.38 [-20.96, 4.15] | -2.88 ± 6.48 [-15.62, 9.87] |  |  |  |  |
| **(i) LDL/HDL ratio** |  |  |  |  |  |  |  |
| Baseline (0W) | 1.92 ± 0.03 [1.87, 1.98] | 1.92 ± 0.03 [1.87, 1.98] | 1.93 ± 0.03 [1.87, 1.99] | Observed values | <0.001 | 0.105 | 0.196 |
| Week 8 | 1.93 ± 0.03 [1.88, 1.99] | 1.85 ± 0.03 [1.80, 1.91] | 1.87 ± 0.03 [1.82, 1.93] |  |  |  |  |
| Week 16 | 1.89 ± 0.03 [1.84, 1.95] | 1.78 ± 0.03 [1.73, 1.84] | 1.86 ± 0.03 [1.80, 1.92] |  |  |  |  |
| Δ Week 8 (change from baseline) | 0.01 ± 0.04 [-0.06, 0.08] | -0.07 ± 0.04 [-0.14, 0.00] | -0.06 ± 0.04 [-0.13, 0.01] | Changes from baseline | 0.028 | 0.112 | 0.527 |
| Δ Week 16 (change from baseline) | -0.03 ± 0.04 [-0.10, 0.04] | -0.14 ± 0.04 [-0.21, -0.07] | -0.08 ± 0.04 [-0.15, 0.00] |  |  |  |  |
| **(j) Fasting glucose (mg/dL)** |  |  |  |  |  |  |  |
| Baseline (0W) | 89.74 ± 0.66 [88.44, 91.04] | 89.76 ± 0.66 [88.46, 91.05] | 89.72 ± 0.68 [88.39, 91.05] | Observed values | <0.001 | 0.326 | 0.463 |
| Week 8 | 90.40 ± 0.67 [89.09, 91.71] | 91.42 ± 0.66 [90.13, 92.71] | 92.35 ± 0.68 [91.01, 93.69] |  |  |  |  |
| Week 16 | 91.68 ± 0.66 [90.38, 92.98] | 91.40 ± 0.66 [90.11, 92.69] | 92.63 ± 0.69 [91.27, 93.99] |  |  |  |  |
| Δ Week 8 (change from baseline) | 0.44 ± 0.83 [-1.19, 2.08] | 1.66 ± 0.82 [0.05, 3.28] | 2.63 ± 0.85 [0.96, 4.30] | Changes from baseline | 0.210 | 0.302 | 0.368 |
| Δ Week 16 (change from baseline) | 1.94 ± 0.83 [0.32, 3.57] | 1.65 ± 0.82 [0.03, 3.26] | 2.91 ± 0.86 [1.22, 4.60] |  |  |  |  |
| **(k) HbA1c (%)** |  |  |  |  |  |  |  |
| Baseline (0W) | 5.55 ± 0.02 [5.52, 5.58] | 5.55 ± 0.02 [5.52, 5.58] | 5.55 ± 0.02 [5.52, 5.58] | Observed values | <0.001 | 0.740 | 0.330 |
| Week 8 | 5.48 ± 0.02 [5.45, 5.51] | 5.45 ± 0.02 [5.42, 5.48] | 5.48 ± 0.02 [5.44, 5.51] |  |  |  |  |
| Week 16 | 5.46 ± 0.02 [5.43, 5.49] | 5.48 ± 0.02 [5.45, 5.51] | 5.49 ± 0.02 [5.46, 5.52] |  |  |  |  |
| Δ Week 8 (change from baseline) | -0.07 ± 0.02 [-0.11, -0.04] | -0.11 ± 0.02 [-0.15, -0.07] | -0.08 ± 0.02 [-0.12, -0.04] | Changes from baseline | 0.293 | 0.728 | 0.139 |
| Δ Week 16 (change from baseline) | -0.09 ± 0.02 [-0.13, -0.05] | -0.07 ± 0.02 [-0.11, -0.03] | -0.06 ± 0.02 [-0.10, -0.03] |  |  |  |  |
| **Legend:** Clinic systolic blood pressure (SBP), clinic diastolic blood pressure (DBP), serum 8-OHdG, hs-CRP, TC, LDL-C, HDL-C, TG, LDL/HDL ratio, fasting plasma glucose, and HbA1c for the NI, HC, and HC+MILK groups at baseline, week 8, week 16, and as changes from baseline. Values are LS mean ± SE (95% CI). P-values are from linear mixed models with fixed effects for group, time, and group×time. Tukey-adjusted post-hoc comparisons were not performed because the group×time interaction was not significant. NI, no intake; HC, high-cacao chocolate; HC+MILK, high-cacao chocolate plus milk-protein beverage; SBP, systolic blood pressure; DBP, diastolic blood pressure; 8-OHdG, 8-hydroxy-2'-deoxyguanosine; hs-CRP, high-sensitivity C-reactive protein; TC, total cholesterol; LDL-C, low-density lipoprotein cholesterol; HDL-C, high-density lipoprotein cholesterol; TG, triglycerides; HbA1c, hemoglobin A1c. | | | | | |  |  |
