# Supplementary material for: Effects of daily intake of cacao polyphenols, alone or with milk protein, on skeletal muscle mass in community-dwelling older adults: an exploratory 16-week randomized, open-label, parallel-group trial
Source: BMC Nutr. 2026 Aug 3;12:229. doi: 10.1186/s40795-026-01445-7 (PMC13721333; doi:10.1186/s40795-026-01445-7)
Supplement: Supplementary file 4 [file 40795_2026_1445_MOESM4_ESM.docx]

| **Table S3. Step counts (steps/day) at baseline, week 8, and week 16.** | |  |  |  |  |  |  |
| --- | --- | --- | --- | --- | --- | --- | --- |
| **Time** | **NI n=76** | **HC n=72** | **HC+MILK n=71** | **P-value** | | | |
|  |  |  |  |  | Time | Group | Time×Group |
| **Steps (steps/day)** |  |  |  |  |  |  |  |
| Baseline (0W) | 6592.01 ± 2887.88 | 7110.14 ± 3959.48 | 7114.62 ± 3638.10 | Observed values | 0.005 | 0.536 | 0.818 |
| Week 8 | 6807.18 ± 3350.17 | 7126.87 ± 3734.63 | 7649.16 ± 3902.59 |  |  |  |  |
| Week 16 | 6303.41 ± 3100.28 | 6709.32 ± 3509.19 | 6922.25 ± 4593.20 |  |  |  |  |
| Δ Week 8 (change from baseline) | 234.01 ± 2125.53 | 16.73 ± 1973.64 | 534.54 ± 2637.59 | Changes from baseline | 0.001 | 0.627 | 0.741 |
| Δ Week 16 (change from baseline) | -288.61 ± 2335.49 | -400.81 ± 3032.82 | -185.51 ± 2925.08 |  |  |  |  |
| **Legend:** Step counts (steps/day) at baseline, week 8, and week 16, and changes from baseline (Δ) at weeks 8 and 16 for the NI, HC, and HC+MILK groups. Values are mean ± SD (n). P-values are from linear mixed models with fixed effects for group, time, and time×group. Tukey-adjusted post-hoc comparisons were not performed because the time×group interaction was not significant. Step counts were averaged over a 3-day monitoring period at each time point. NI, no intake; HC, high-cacao chocolate; HC+MILK, high-cacao chocolate plus milk-protein beverage. | | | | | | | |
