# Supplementary material for: Effects of daily intake of cacao polyphenols, alone or with milk protein, on skeletal muscle mass in community-dwelling older adults: an exploratory 16-week randomized, open-label, parallel-group trial
Source: BMC Nutr. 2026 Aug 3;12:229. doi: 10.1186/s40795-026-01445-7 (PMC13721333; doi:10.1186/s40795-026-01445-7)
Supplement: Supplementary file 5 [file 40795_2026_1445_MOESM5_ESM.docx]

| **Table S4. Dietary intake at baseline and week 16 estimated using the BDHQ.** | | |  |  |  |  |
| --- | --- | --- | --- | --- | --- | --- |
| **Time** | **NI n=76** | **HC n=72** | **HC+MILK n=71** | **P-value** | | |
|  |  |  |  | Time | Group | Time×Group |
| **(a) BDHQ Energy (kcal/day)** |  |  |  |  |  |  |
| Baseline (0W) | 1610.94 ± 27.27 [1557.34, 1664.53] | 1620.75 ± 28.02 [1565.68, 1675.81] | 1615.46 ± 28.20 [1560.04, 1670.88] | 0.444 | 0.594 | 0.387 |
| Week 16 | 1665.67 ± 27.27 [1612.08, 1719.27] | 1599.31 ± 28.02 [1544.25, 1654.38] | 1634.50 ± 28.61 [1578.27, 1690.72] |  |  |  |
| **(b) BDHQ Protein (g/day)** |  |  |  |  |  |  |
| Baseline (0W) | 66.84 ± 1.32 [64.24, 69.44] | 67.33 ± 1.36 [64.65, 70.00] | 66.79 ± 1.37 [64.10, 69.48] | 0.300 | 0.252 | 0.156 |
| Week 16 | 70.83 ± 1.32 [68.23, 73.43] | 66.27 ± 1.36 [63.60, 68.95] | 67.29 ± 1.39 [64.56, 70.01] |  |  |  |
| **(c) BDHQ Fat (g/day)** |  |  |  |  |  |  |
| Baseline (0W) | 52.27 ± 1.05 [50.19, 54.34] | 52.57 ± 1.08 [50.44, 54.70] | 52.34 ± 1.09 [50.19, 54.48] | 0.262 | 0.719 | 0.539 |
| Week 16 | 52.26 ± 1.05 [50.19, 54.33] | 50.26 ± 1.08 [48.13, 52.39] | 51.69 ± 1.11 [49.52, 53.87] |  |  |  |
| **(d) BDHQ Carbohydrate (g/day)** |  |  |  |  |  |  |
| Baseline (0W) | 207.15 ± 4.30 [198.70, 215.60] | 208.88 ± 4.41 [200.20, 217.55] | 208.18 ± 4.44 [199.44, 216.92] | 0.182 | 0.839 | 0.653 |
| Week 16 | 214.93 ± 4.30 [206.48, 223.38] | 209.06 ± 4.41 [200.38, 217.74] | 214.60 ± 4.51 [205.74, 223.46] |  |  |  |
| **(e) BDHQ Total dietary fiber (g/day)** |  |  |  |  |  |  |
| Baseline (0W) | 12.19 ± 0.27 [11.66, 12.73] | 12.26 ± 0.28 [11.71, 12.81] | 12.11 ± 0.28 [11.56, 12.67] | 0.061 | 0.998 | 0.870 |
| Week 16 | 12.59 ± 0.27 [12.06, 13.13] | 12.55 ± 0.28 [12.00, 13.10] | 12.70 ± 0.29 [12.14, 13.27] |  |  |  |
| **(f) BDHQ Salt (sodium chloride) equivalent (g/day)** |  |  |  |  |  |  |
| Baseline (0W) | 9.91 ± 0.17 [9.58, 10.24] | 9.97 ± 0.17 [9.63, 10.31] | 9.95 ± 0.17 [9.61, 10.29] | 0.980 | 0.278 | 0.157 |
| Week 16 | 10.29 ± 0.17 [9.96, 10.62] | 9.72 ± 0.17 [9.38, 10.06] | 9.83 ± 0.18 [9.48, 10.17] |  |  |  |
| **Legend:** Energy, protein, fat, carbohydrate, total dietary fiber, and sodium chloride equivalent intake estimated using the Brief-type self-administered Diet History Questionnaire (BDHQ) for the NI, HC, and HC+MILK groups. Values are LS mean ± SE with 95% confidence intervals in brackets.  P-values are from linear mixed models with fixed effects for group, time, and group×time. Tukey-adjusted post-hoc comparisons were not performed because the group×time interaction was not significant. NI, no intake; HC, high-cacao chocolate; HC+MILK, high-cacao chocolate plus milk-protein beverage; BDHQ, Brief-type self-administered Diet History Questionnaire. | | | | | |  |
