# Supplementary material for: Effects of daily intake of cacao polyphenols, alone or with milk protein, on skeletal muscle mass in community-dwelling older adults: an exploratory 16-week randomized, open-label, parallel-group trial
Source: BMC Nutr. 2026 Aug 3;12:229. doi: 10.1186/s40795-026-01445-7 (PMC13721333; doi:10.1186/s40795-026-01445-7)
Supplement: Supplementary file 6 [file 40795_2026_1445_MOESM6_ESM.docx]

**Table S5. Baseline-adjusted standardized mean differences for body-composition outcomes at week 8 and week 16.**

| **Time** | **HC − NI** | **HC+MILK − NI** | **HC+MILK − HC** |
| --- | --- | --- | --- |
| **(a) Weight (kg)** |  |  |  |
| Week 8 | 0.215 ± 0.165 [-0.110, 0.540] | 0.484 ± 0.167 [0.154, 0.814] | 0.269 ± 0.168 [-0.063, 0.601] |
| Week 16 | 0.307 ± 0.165 [-0.019, 0.633] | 0.509 ± 0.168 [0.178, 0.841] | 0.202 ± 0.169 [-0.130, 0.535] |
| **(b) BMI (kg/m²)** |  |  |  |
| Week 8 | 0.167 ± 0.166 [-0.160, 0.493] | 0.435 ± 0.167 [0.105, 0.764] | 0.268 ± 0.169 [-0.065, 0.600] |
| Week 16 | 0.257 ± 0.166 [-0.070, 0.584] | 0.500 ± 0.168 [0.168, 0.831] | 0.242 ± 0.169 [-0.091, 0.576] |
| **(c) Percent body fat (%)** |  |  |  |
| Week 8 | -0.284 ± 0.166 [-0.612, 0.044] | -0.132 ± 0.167 [-0.461, 0.197] | 0.152 ± 0.169 [-0.181, 0.486] |
| Week 16 | -0.199 ± 0.166 [-0.526, 0.128] | -0.099 ± 0.167 [-0.428, 0.229] | 0.099 ± 0.169 [-0.234, 0.433] |
| **(d) Soft lean mass (SLM) (kg)** |  |  |  |
| Week 8 | 0.406 ± 0.166 [0.078, 0.733] | 0.461 ± 0.168 [0.130, 0.792] | 0.056 ± 0.169 [-0.278, 0.389] |
| Week 16 | 0.556 ± 0.167 [0.227, 0.886] | 0.615 ± 0.169 [0.282, 0.948] | 0.059 ± 0.169 [-0.274, 0.392] |
| **(e) Fat-free mass (FFM) (kg)** |  |  |  |
| Week 8 | 0.402 ± 0.166 [0.074, 0.729] | 0.460 ± 0.168 [0.129, 0.791] | 0.059 ± 0.169 [-0.275, 0.392] |
| Week 16 | 0.537 ± 0.167 [0.207, 0.866] | 0.620 ± 0.169 [0.287, 0.953] | 0.084 ± 0.169 [-0.250, 0.417] |
| **(f) Skeletal muscle mass (SMM) (kg)** |  |  |  |
| Week 8 | 0.428 ± 0.166 [0.100, 0.756] | 0.511 ± 0.168 [0.179, 0.843] | 0.083 ± 0.169 [-0.250, 0.417] |
| Week 16 | 0.519 ± 0.167 [0.190, 0.849] | 0.637 ± 0.169 [0.304, 0.971] | 0.118 ± 0.169 [-0.216, 0.451] |
| **(g) Skeletal muscle index (SMI) (kg/m²)** |  |  |  |
| Week 8 | 0.444 ± 0.166 [0.115, 0.772] | 0.441 ± 0.168 [0.110, 0.771] | -0.003 ± 0.169 [-0.337, 0.330] |
| Week 16 | 0.495 ± 0.167 [0.166, 0.824] | 0.541 ± 0.168 [0.209, 0.873] | 0.046 ± 0.169 [-0.287, 0.380] |
| **(h) ECW/TBW (ratio)** |  |  |  |
| Week 8 | -0.024 ± 0.167 [-0.354, 0.305] | -0.119 ± 0.169 [-0.452, 0.214] | -0.095 ± 0.169 [-0.428, 0.239] |
| Week 16 | 0.255 ± 0.168 [-0.076, 0.585] | 0.043 ± 0.169 [-0.290, 0.376] | -0.212 ± 0.169 [-0.546, 0.122] |

**Legend:** Baseline-adjusted standardized mean differences for the HC − NI, HC+MILK − NI, and HC+MILK − HC contrasts at week 8 and week 16. At each follow-up time point, adjusted group means were estimated using an analysis-of-covariance model including group and the corresponding baseline value; contrasts were standardized using the residual standard deviation of the model. Values are standardized mean difference ± SE with 95% confidence intervals in brackets, calculated using emmeans::eff_size. Contrasts are presented as the former group minus the latter; positive values indicate a higher adjusted outcome in the former group. NI, no intake; HC, high-cacao chocolate; HC+MILK, high-cacao chocolate plus milk-protein beverage.
